# Performance Enhancement of the Polarimetric Fibre Optical Current Sensor at JET Using Polarisation Optimisation

**DOI:** 10.3390/s24020555

**Published:** 2024-01-16

**Authors:** Andrei Gusarov, Perry Beaumont, Paula Siren, JET Contributors

**Affiliations:** 1 SCK CEN Belgian Nuclear Research Centre, 2400 Mol, Belgium; 2 The Joint European Torus (JET), Culham Centre for Fusion Energy (CCFE), Culham Science Centre, Abingdon OX14 3EB, UK; perry.beaumont@ukaea.uk (P.B.); paula.siren@ukaea.uk (P.S.)

**Keywords:** fibre optics current sensor (FOCS), polarimetry, polarisation adjustment, plasma current, tokamak, joint European torus (JET)

## Abstract

To achieve optimal operation of the polarimetry-based FOCS, the light polarisation state at the input of the sensing fibre part must be close to a linear one. In the case of a FOCS deployed on a tokamak, the Joint European Torus (JET) in the present work, the long fibre optics link between the laser source and the sensing fibre modifies the polarisation in an unpredictable way, making it unclear which source polarisation state is to be set. A method for performing the necessary polarisation adjustment in a systematic way is proposed based on the FOCS analysis. The method requires performing data acquisition at two different input polarisations. Based on these measurements, the optimal laser source polarisation can be found. The method was experimentally verified using laboratory set-up and then successfully demonstrated with the FOCS installed at JET.

## 1. Introduction

Stable operation of the magnetic fusion devices, tokamaks, depends on the generation of current in the plasma. As a result, precise knowledge of this current is a very important safety requirement. From the beginning of the tokamak research, plasma current measurements are based on inductive type sensors of various types: Rogowski coils, pick-up coils, saddle loops, etc. [1]. Although those sensors have different forms, they share a common feature that the signal is proportional to the time derivative of the magnetic flux through the sensor loop and reconstruction of the current involves the integration step. For plasma operation with a duration of several tens of minutes advanced integration techniques allow for the problem to be solved, as it was demonstrated at Tore-Supra [2] and KSTAR [3,4]. However, even for the advanced techniques, non-linear signal drifts remain a significant challenge [5] and it is not clear if extension for longer operation times is possible. Evidently, the need for integration can be a problem for the steady-state tokamak operation regime, which will be implemented in future energy generation installations. Another concern is related to the presence spurious currents generated by strong nuclear radiation during burning plasma operation. [6].

In this situation, implementation of non-inductive sensors for plasma current measurements is regarded as an attractive option. Currently, the Hall sensors [7] and the Fibre Optics Current Sensor (FOCS) [8] are the most promising candidates and they will be implemented at ITER. The ITER FOCS system will use optical fibres placed around the Vacuum Vessel (VV) to measure the ITER toroidal current based on the polarisation detection approach [9].

**Figure 1:**
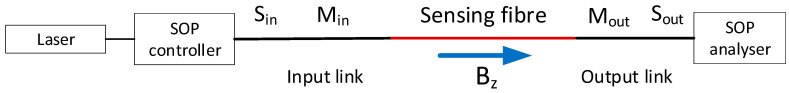
FOCS schematic representation. S_in_, S_out_ are the Stokes vectors for input and output light, M_in_, M_out_ are the Mueller matrices for the input and output links, respectively. The blue arrow indicates that the component of magnetic field B_z_ aligned with the fibre axis.

In the future burning plasma machines, including ITER, the sensing fibre will be subject to a harsh environment. In particular, intense nuclear radiation field and elevated temperatures, with the loads significantly exceeding levels usually considered for FOCS in industrial applications, can create significant perturbations. Thanks to the use of the polarisation detection approach adopted for ITER FOCS, the system is expected to be rather tolerant to the most common effect of radiation-induced transmission degradation [9]. However, the data-acquisition electronics cannot withstand the radiation levels near the VV and must be placed at a sufficient distance. Therefore, optical links with a length in a range of ~100 m are inevitable. Figure 1 shows schematics of the tokamak FOCS operating in transmission.

The experience of using FOCS at big fusion facilities is rather limited [10,11]. The presence of long fibre links is a significant challenge for FOCS, because these links may create unpredictable polarisation perturbations. From the point of view of addressing this and other challenges, performing measurements at JET is the best available option. JET provides an environment, which is fully representative for future burning plasma installation, both in terms of plasma currents and radiation loads. For example, during the D-T operation it is possible to achieve on the external surface of the JET vacuum vessel neutron fluxes of ~10^10^ n/cm^2^/s [12]. Similar values are expected for ex-vessel sensors at ITER during D-T operation [13].

Based on this assessment, the FOCS system was installed on JET. It was used to perform current measurement during numerous campaigns, including the D-T, with some results already reported [14]. The FOCS at JET uses the low-birefringence (Low-Bi) spun fibre for the sensing part, placed on the vacuum vessel. Spun fibres are produced by spinning the preform during the fibre drawing stage. For the JET FOCS a low birefringence preform was used with the spun period much shorter than the linear birefringence beat length of a standard fibre drawn from the same preform. As a result, the spun fibre has a low birefringence combined with a low sensitivity to external perturbations.

To achieve a high performance operation of FOCS, the light polarisation at the input of the sensing fibre must be close to linear [14]. However, the long optical fibre link inserted after the laser modifies the light polarisation state. The laser source polarisation must be particularly adjusted and, in general, this adjustment may vary with time. In the present work, we address the problem of the input polarisation adjustment and its effect on the current measurement accuracy.

## 2. FOCS Installation at JET

A detailed description of the JET FOCS was already given in [14]. FOCS operation relies on the Faraday effect in optical fibres, rotation of the polarisation axis induced by magnetic field aligned with the fibre’s propagation direction. The JET FOCS optical scheme is based on the polarisation rotation detection. The optical scheme is shown in Figure 1. The laser LS5-c-29B-20-NM operates at 1546.5 nm with a 20 mW power. The SOP adjustments are performed with the Deterministic Polarisation Controller DPC5500, which produces a stable outpot SOP, independent of the input light polarisation. The SOP accuracy is ±0.25° on the Poincaré Sphere. A Fast Inline Polarimeter IPM5300 is used to analyse the output SOP. All equipment is from Thorlabs Inc., USA In the ideal case, when the birefringence of the components is negligible, the polarisation rotation angle θ as a result of the Faraday effect is proportional to the enclosed current I:

(1) $$\theta =V_{c}I,$$

where $V_{c}$ the Verdet constant.

**Figure 2:**
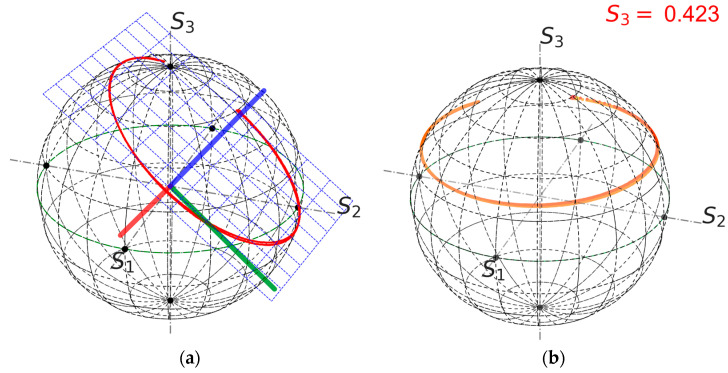
Example of definition of the new reference plane, data from shot 91962. (**a**) Initial data with the basis plane, which is represented by blue dotted lines; the thick blue line represents the axis orthogonal to the plane and two other axes represented by the red and green lines; (**b**) representation of the data in the rotated system of coordinates. For the rotated data, mean (*S*_3_) = 0.423.

A convenient way to explain FOCS operation is to use the Poincaré Sphere (PS). Each point on the sphere corresponds to a particular SOP, which is defined by the three-component Stokes vector $S={\left(S_{1},S_{2},S_{3}\right)}^{T}$. In the ideal case, polarisation rotation induced by the Faraday effect corresponds to an arc of a circle parallel to the equatorial plane [15], and the polarisation rotation angle is equal to the azimuth change. In the real situation, the output FOCS link changes the polarisation state. In particular the linear birefringence results in a tilt of the arc, as it is shown in Figure 2a. This means that the azimuth change is not equal to the polarisation rotation angle. A way to retrieve the rotation angle is to change the reference frame so that the SOP trace is located in a plane parallel to the equatorial one [11]. As soon as the experimental data contain errors, the problem has no exact solution. One possible approach consists of fitting a plane through the measurement points [14]. This procedure defines the 3 × 3 rotation matrix *T* so that the Stokes parameters in the new basis are computed as:

(2) $$S^{c}=TS.$$

In the new reference frame, the Faraday rotation angle is obtained in a simple way:

(3) $$\theta \left(t\right)=0.5\cdot \left(atan\;(S_{2}^{c}\left(t\right)/S_{1}^{c}(t))-atan\;(S_{2}^{c}\left(0\right)/S_{1}^{c}(0))\right),\;0\leq \theta \leq \pi$$

here ${S_{1}^{c}(t),\;S}_{2}^{c}(t)$ are the Stokes components in the new basis at a moment *t*.

This approach is illustrated in Figure 2 using experimental data. The blue axis in Figure 2a is the basis vector normal to the plane containing the experimental points. Two other basis vectors are defined arbitrary to preserve the handedness. In the rotated frame the trace is longitudinal and is shifted with respect to the equatorial plane with mean (*S*_3_) = 0.423, which means that the input polarisation is an elliptic state.

The experimental data in Figure 2 are referred as “data from shot 91962”. At JET, a regular plasma operation duration is ~80 s and it is separated from another plasma operation by a much longer interval, at least an hour-scale. These plasma operations are called “shots” or “pulses”. At the JET start-up in 1983, a shot counter was set. This counter is increased by one for each attempt of plasma operation (successful or not) and for preparations of plasma operation, when various tokamak systems are tested before starting plasma discharge, for example “dry runs”. Each plasma shot is defined by a specific set of machine parameters and the counter provides a convenient way of identifying such a set. In December 2024, the counter showed 105929.

Using elliptical polarisation for the measurements is not desirable because it decreases the measurement accuracy. According to the specification of the IPM5300, the device has a measurement accuracy of ∆ε=±0.25° on the PS for averaging times above 1 ms. This accuracy can be represented as a spreading of measurement points within a circle centred around the actual SOP. In case of a linear input polarisation the Faraday rotation, after applying the rotational transformation, corresponds to a displacement along the equator. According to Equation (1) the Faraday rotation angle depends on the applied current and the Verdet constant. For the silica-based fibre used in JET FOCS the Verdet constant is $V_{c}\sim 0.7\;\mu rad/A$. Therefore, the possible error is defined as $∆I=0.5∆\varepsilon /V_{c}=\pm 3.12\;kA$. This value represents the minimal achievable measurement error when using IPM5300. For the case of an elliptical input polarisation Faraday rotation results in a trace along a parallel at an χ degrees latitude from the equator. The length of the arc is reduced by a factor $cos\left(\chi \right)$ and the corresponding measurement error is $∆I/cos\left(\chi \right)=∆I/\sqrt{1-S_{3}^{2}}$. It grows infinitely when the latitude approaches a pole $S_{3}\to \pm 1$. Physically, this means that for the polarimetry scheme the optimal performance is achieved with a linear input polarisation, while the polarisation is not detectable when the input polarisation is circular.

As a result of the polarisation effect of the input link fibre, the output light of the polarisation controller DPC-5500 should be, in general, adjusted to a specific elliptic polarisation state, which after propagation is converted into a linear polarisation (*S*_3_ = 0) at the input of the sensing fibre. This polarisation adjustments need to be carried out at the start of operation and then repeated on a regular basis to compensate for large SOP drifts. How often the latter need to be carried out depends on the stability of both the polarization controller and the optical fibre link between the controller and the input of the sensing fibre. It follows from the comparison of FOCS measurements in a stable laboratory environment and at JET that the overall set-up stability is defined by the optical fibre link. The latter depends on the environmental temperature in the tokamak building, which changes during operation, and stresses related to vibrations. These instabilities are the main reason why polarization adjustments are required. 

The polarisation characteristics of the link fibre are unknown. Therefore, in the JET experiment, the output SOP of the DPC-5500 is defined by a trial-and-error method: the ellipticity and the azimuth of the controller output are modified to obtain a trace on the Poincaré sphere with the radius close to one. Taking into account that the effect of deviation from the linear SOP is proportional to $1/\sqrt{1-S_{3}^{2}}$, the polarisation states resulting in abs(*S*_3_) < 0.1 are considered as acceptable. To achieve such a result a few trials are usually sufficient. On the other hand, satisfying a more favourable requirement abs(*S*_3_) << 0.1 is not feasible, because it would require many more attempts. Additionally, a reduction in the number of the trials is desirable.

## 3. Analysis of the FOCS Polarisation Adjustment

For the time being, we neglect the influence of the output link. A reciprocal optical system containing any number of retardation plates and rotators is optically equivalent to a system containing only two elements: a linear retarder and a rotator [16]. The input and output links can be represented in this way. When the spun period is much shorter than the linear beat length of an equivalent non-spun fibre, the sensing spun fibre can be approximated as an ideal rotator, where only circular birefringence related to the spun and the Faraday effect are present and is described by a rotation matrix. Therefore, if the SOP at the exit of the equivalent linear retarder is a linear polarisation, it will be a linear state at the entry of the SOP analyser and vice versa.

The Mueller matrix of a retarder $\delta \in \left[0;2\pi \right]$ rotated by an angle $\theta \in \left[0;\pi \right]$ is defined as [17]:

(4) $$M\left(\delta ,\theta \right)=\left[\begin{array}{cccc} 1 & 0 & 0 & 0\\ 0 & {cos}^{2}2\theta +\cos\;\delta \;{sin}^{2}2\theta & 0.5\left(1-\cos\;\delta \right)\sin\;4\theta & -\sin\;\delta \;\sin\;2\theta \\ 0 & 0.5\left(1-\cos\;\delta \right)\sin\;4\theta & {sin}^{2}2\theta +\cos\;\delta \;{cos}^{2}2\theta & \sin\;\delta \;\cos\;2\theta \\ 0 & \sin\;\delta \;\sin\;2\theta & -\sin\;\delta \;\cos\;2\theta & \cos\;\delta \end{array}\right]$$

The third component of the Stokes vector at the exit of the equivalent retarder is defined as:

(5) $$A_{3}=MS=M_{3,1}S_{1}+M_{3,2}S_{2}+M_{3,3}S_{3},$$

where $S_{j},\;j=1,\;2,\;3$ are components of the Stokes vector S, and M is the Mueller matrix of the rotated retarder. As it was noted, the rest of the equivalent optical scheme is a rotator. The Mueller matrix of a rotator $\varphi \in \left[0;\pi \right]$:

(6) $$R\left(\varphi \right)=\left[\begin{array}{cccc} 1 & 0 & 0 & 0\\ 0 & \cos\;2\varphi & \sin\;2\varphi & 0\\ 0 & -\sin\;2\varphi & \cos\;2\varphi & 0\\ 0 & 0 & 0 & 1 \end{array}\right]$$

It is obvious from Equation (6) that rotation does not influence the $S_{3}$ component of the Stokes vector. Therefore, the value measured by the SOP analyser is the same as that at the entry of the sensing fibre.

Now we need to take into account the output link. It is also equivalent to a combination of a rotated linear retarder and a rotator. The effect of the retarder is to rotate the FOCS trace on the Poincaré sphere [18]. If we apply the rotational transformation of [14], the $S_{3}$ value will be the same as the input of the equivalent output retarder.

The conclusion of this discussion is that the two parameters, the phase delay and the orientation of the equivalent input retarder, which are required to know an input polarisation, which will result in a linear state at the input of the sensing fibre, can be found from the SOP analyser data. It may be useful to note that the azimuth of this linear state is not defined, but this has no influence on the optimisation because any linear polarisation is acceptable.

To find the two parameters, two measurements should be sufficient. They can be found by numerically solving the system:

(7) $$A_{3}^{i}=M_{3,*}S^{i}=M_{3,1}S_{1}^{i}+M_{3,2}S_{2}^{i}+M_{3,3}S_{3}^{i},\;i=1,\;2$$

where $S_{j}^{i},\;j=1,\;2,\;3$ are components of the Stokes vectors $S^{i}$, describing two arbitrary different input polarisation states, M is the Mueller matrix of the rotated retarder, and $A_{3}^{i}$ is the third component of the measured Stokes vector.

When the M-components are defined, it is possible to find a Stokes vector $S^{3}$ of the SOP controller, which will give a linear (optimal) SOP $A^{3}$ at the input of the sensing fibre, by solving the equation:

(8) $$A_{3}^{3}=M_{3,1}S_{1}^{3}+M_{3,2}S_{2}^{3}+M_{3,3}S_{3}^{3}=0$$

The components of Stokes vectors are defined by ellipticity χϵ−π4,π4, and azimuth $\psi \epsilon \left[0,\pi \right]$:

(9) $$S_{1}=\cos\;2\chi \cos\;2\psi ,\;\;S_{2}=\cos\;2\chi \sin\;2\psi ,\;\;S_{3}=\sin\;2\chi$$

Therefore, Equation (8) has two independent variables, the ellipticity and the azimuth, and thus has multiple solutions. This means that further optimisation can be carried out, for example, to obtain a specific polarisation. Another possibility is to restrict input polarisation states, for example, to linear. The latter possibility was further used because this approach is considered for future FOCS installation at ITER.

## 4. Laboratory Verification of the Polarisation Adjustment Approach

**Figure 3:**
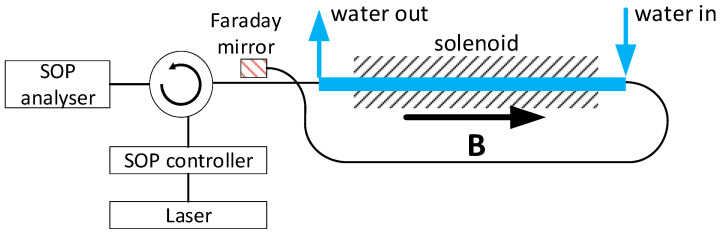
Magnetic testbed. Laser—1550 nm, LS5-c-29B-20-NM, Thorlabs; SOP controller—DPC 5500, Thorlabs; SOP Analyser—IPM 5300, Thorlabs. The dashed area represents the solenoid.

In order to verify the proposed approach, we performed laboratory tests using magnetic testbed at SCK CEN, Mol, Belgium, schematically shown in Figure 3. The fibre to be tested is inserted into a long solenoid assembled from sixteen small solenoids made of a copper wire. The length of a small solenoid is 17 cm; thus, the total length of the fibre in the magnetic field is 272 cm. The fibre makes a closed loop around the solenoids. According to the Ampere theorem, the total polarisation rotation is proportional to the enclosed current, which is the product of the current on the number of the wire turns. To perform measurements the set-up is equipped with Sorensen DCS150-20E units. The sixteen solenoids are divided into four group with four units each. Each group of four solenoids is powered by one Sorensen DCS150-20E. The maximal stabilised current which can be obtained on one solenoid is 25 kA, which corresponds to the total current of 400 kA.

During operation with the maximal current the temperature of solenoids grows as a result of ohmic heating at a ~0.7°/s rate. The maximal temperature is limited to ~80 °C to avoid degradation of electric insulation, which limits the operation to ~80 s, after that the system must cool down. At 30% of the maximal current the set-up can operate continuously. Because of the temperature dependence of the Verdet constant temperature control of the fibre is essential for good measurements. This is obtained by flowing water through a plastic tube inserted in the solenoids. In this way, the temperature of the fibre can be stabilised with an accuracy of ±0.1 °C in a range from 10 to 80 °C.

For the measurements, the FOCS configuration operating in reflection with a Faraday mirror (FM) was used. A retracing scheme with a FM allows compensating for reciprocal linear birefringence [19]. Therefore, propagation back and forward through the output link results only in an additional 90° rotation. The analysis of the system operating in transmission is fully applicable and the optimal polarisation controller can be found in the same way.

**Figure 4:**
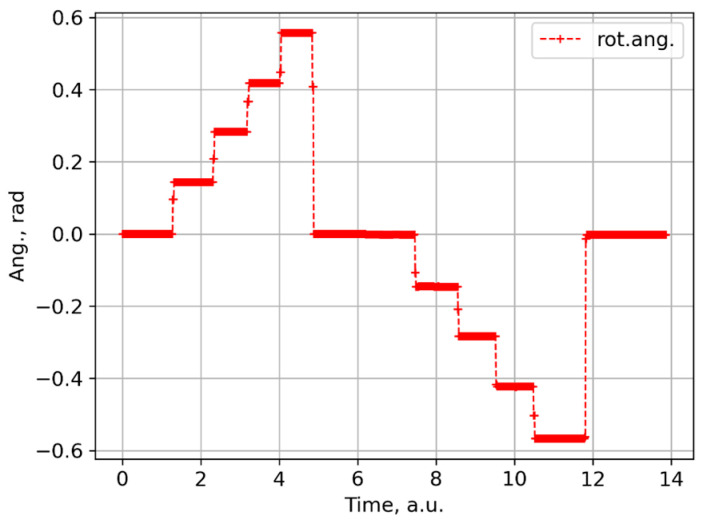
Polarisation rotation angle profile in the laboratory measurements.

**Figure 5:**
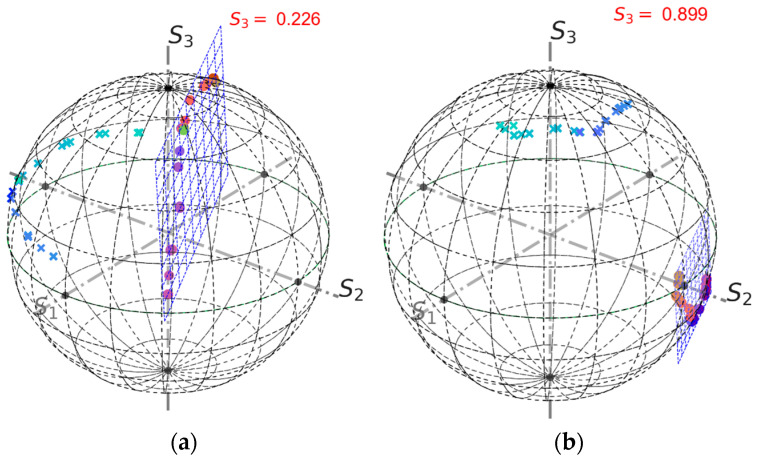
FOCS measurement results on the Poincaré sphere; (**a**,**b**)—measurements with two input polarisations on the SOP controller. Points represent raw data and crosses the same data after rotation transformation. Colour is used to indicate different measurements. The blue grid is the plane fitted to the experimental points. The *S*_3_ values are 0.226 and 0.899, respectively.

**Figure 6:**
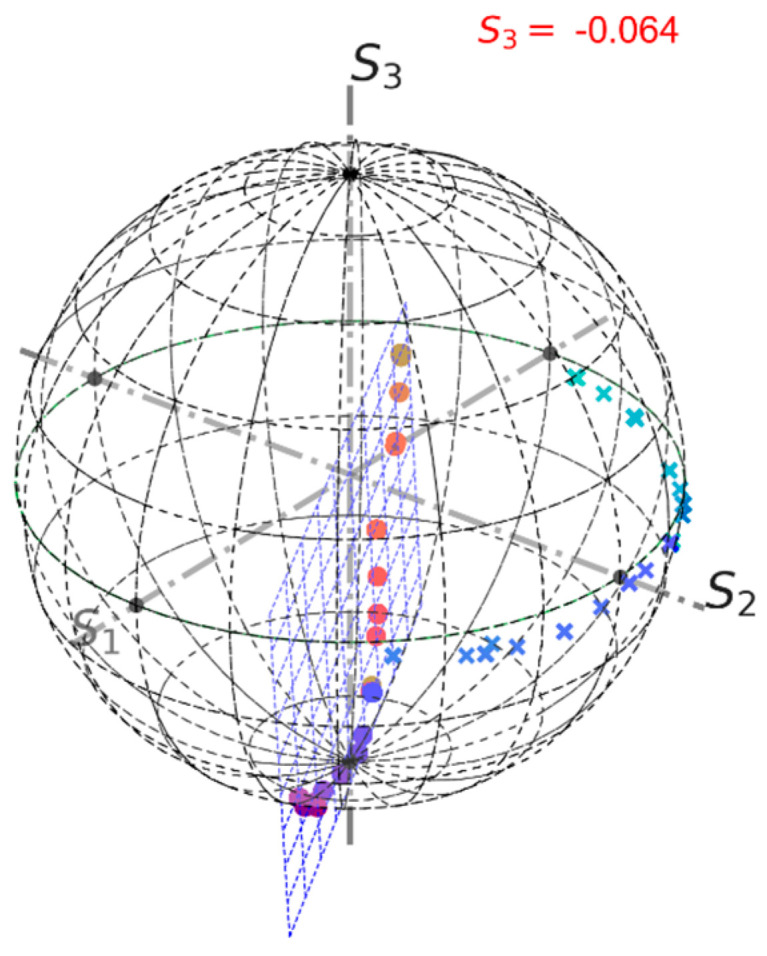
FOCS laboratory measurement results on the Poincaré sphere for the optimised input SOP. Points—represent measurement data; crosses—the same data after rotation transformation; The blue grid is the plane fitted to the experimental points. *S*_3_ = −0.064, which means that the polarisation is close to the optimal.

The power supplies for each group of four solenoids were manually sequentially switched on and then switched off altogether. Then, the current polarity was inverted, and the sequence repeated. The measured rotation angle trace is shown in Figure 4. Each step corresponds to switching a current supply unit. Changing the rotation sign is related to the current polarity change. Figure 5 shows output Stokes parameters for two different input polarisations. Based on these data, the optimal input polarisation was found. Results of a third measurement are shown in Figure 6. The obtained $S_{3}$ value is small, but not exactly zero. This is a consequence of a relatively large measurement error. For example, it can be seen in Figure 5, that for the second measurement points significantly deviate from an arc.

## 5. FOCS Polarisation Adjustment at JET

**Figure 7:**
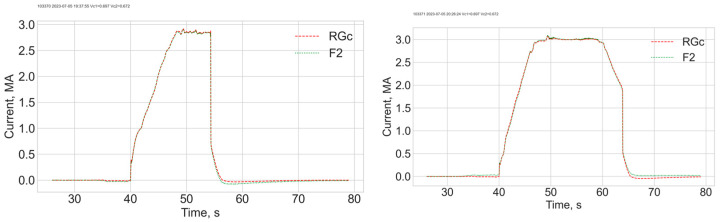
Current profiles for shots 103370 and 103371. F2—FOCS, RGc—reference Rogowski coil.

**Figure 8:**
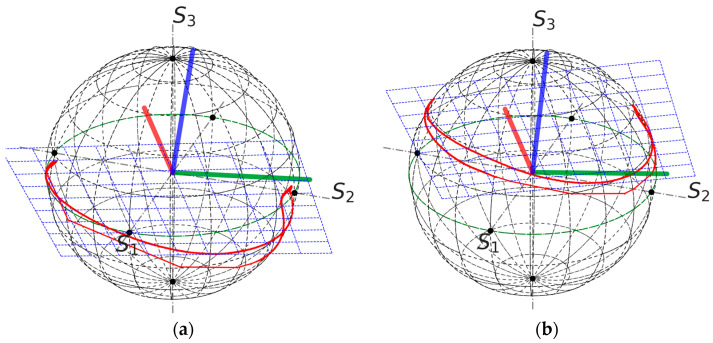
FOCS measurement results on the Poincaré sphere for the shots 103370 (**a**) and 103371 (**b**) when the input polarisation was manually adjusted.

The polarisation adjustment method was subsequently tested in a real tokamak environment, using the FOCS system installed at JET. Two shots, 103370 and 103371, were used in order to obtain data for the polarisation adjustment. The current profiles are shown in Figure 7. The figures show a good agreement between the FOCS and the data of the reference Rogowski coil. The input polarisations were manually adjusted for these shots. Representation of the FOCS measurement results on the Poincaré sphere is shown in Figure 8. Figure 8a,b show the evolution of the Stokes vector during the two shots when the current increases from zero to a maximal value and then decreases back to zero. As a consequence of the polarization adjustments the traces begin at different points on the Poincaré sphere, but otherwise are similar. The difference in the current traces shown in Figure 7 results only in different densities of points along the curves in Figure 8. The latter cannot be shown in Figure 8 because the curve consists of 55,000 measurement points.

**Figure 9:**
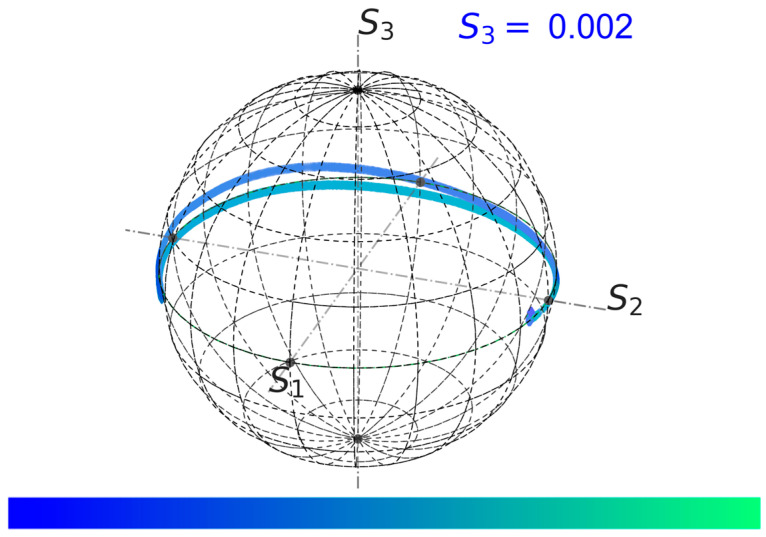
FOCS measurement results on the Poincaré sphere for shot 103372 with the optimised input SOP. The average *S*_3_ = 0.002, which means that the polarisation is close to the optimal. The colour map representation is used to show the shot progress. The dark blue colour corresponds to the start and the light green to the end.

Based on the obtained data, the optimal polarisation for the next shot 103372 was found and set. The FOCS measurement result is shown in Figure 9. A small value of the *S*_3_ parameter was achieved. This figure shows variation in the Stokes vector during the JET plasma operation using the colour map representation.

**Figure 10:**
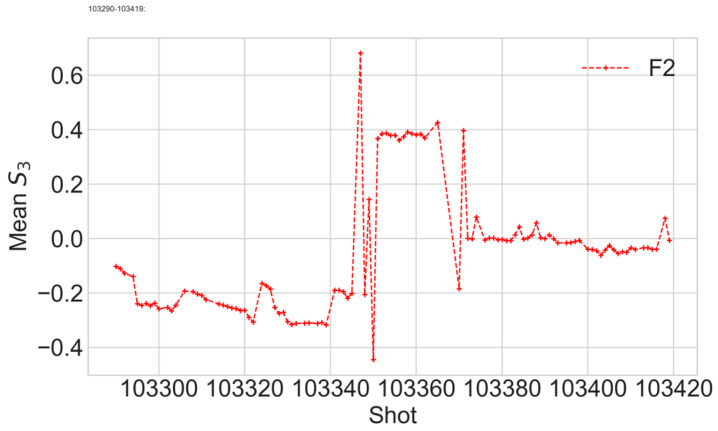
Variation in the mean S_3_ value for shots from 130290 to 103420. Big values for the shots 130345 to 103368 are a consequence of manual changes of the laser polarisation output.

**Figure 11:**
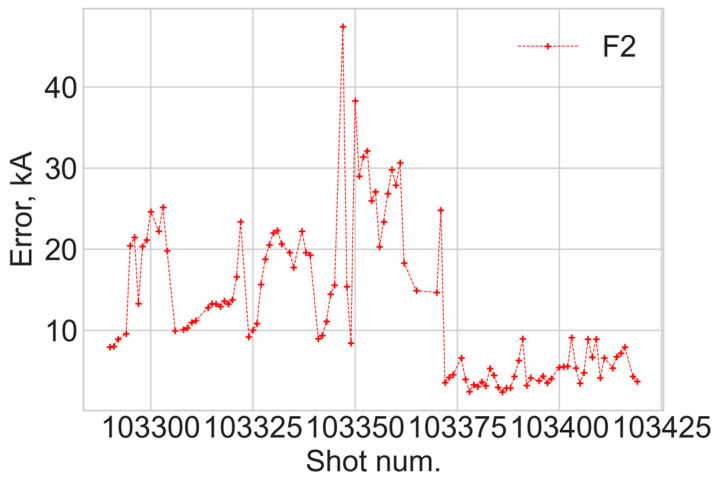
Variation in the current measurement error for shots from 130290 to 103420.

Variation in *S*_3_ over shots from 103290 to 103420 is shown in Figure 10. Before shot 103345, no change in the polarisation was made, and the variations represent the FOCS stability. Then, manual polarisation adjustments were made for shots 103345 to 103371. After that, the input polarisation remained unchanged. After shot 103372, variations in the *S*_3_ mean value are within ±0.05, which means the polarisation at the input of the sensing fibre remains close to linear. It was discussed earlier that the linear polarisation at the input of the sensing fibre is required to obtain the optimal FOCS performance. It is, therefore, useful to compare the *S*_3_ variations with the FOCS measurement error, Figure 11. This error is defined as the mean value of the absolute difference between FOCS current, and the reference current measured by the Rogowski coil.

Using the relative error could be an interesting option for Figure 11, but its realization requires several assumptions. Before the start and after the end of the plasma operation the plasma current is zero, while the FOCS gives a signal fluctuating around the zero, corresponding to currents with an amplitude of several kA, which is defined by the accuracy of the polarimeter, as it is explained in Section 2. As a result, the relative error is infinite. It is possible to circumvent this problem by defining the relative error by only taking into account currents above a certain threshold. As soon as the absolute error is constant, the relative error in this case will depend on both the definition of the threshold, the maximal plasma current and the plasma current profile. For example, for a long flat-top plasma operation, the role of the threshold selection will be less important compared to a triangular current profile with the same maximum value. Therefore, interpretation of the relative error data is rather complicated. On the other hand, the goal of the polarization optimisation is to reduce the absolute error, and Figure 11 demonstrates that this goal is indeed achieved.

## 6. Conclusions

To achieve a high performance of a polarimetry-based FOCS operation, the polarisation of the laser source must be adjusted so that the polarisation state is linear at the input of the sensing fibre. In case of a FOCS system installed in a tokamak environment, this task is complicated by the inevitable presence of a long fibre link between the optical source and the sensing fibre. This optical link modifies the polarisation in an unpredictable way. Therefore, polarisation adjustment, which takes into account unknown properties of the link fibre, is required. A method for performing the adjustment in a systematic way is proposed based on the FOCS analysis. The method requires performing data acquisition at two different input polarisations. Practically this means that only two adjustments allow achieving the optimal polarisation. The first measurement is taken at the present SOP controller settings. Then, the SOP is adjusted, and the second measurement is performed. Based on these two measurements, the optimal SOP is defined, and the optimal state is obtained after the second adjustment. The method was experimentally verified using laboratory set-up and then demonstrated with the FOCS installed at the Joint European Torus (JET) tokamak. It is also experimentally shown that the polarisation optimisation allows for the reduction in measurement error.

## Data Availability

The data presented in this study can be requested from the JET.
